# Skeletal Muscle Extracellular Matrix – What Do We Know About Its Composition, Regulation, and Physiological Roles? A Narrative Review

**DOI:** 10.3389/fphys.2020.00253

**Published:** 2020-03-19

**Authors:** Robert Csapo, Matthias Gumpenberger, Barbara Wessner

**Affiliations:** ^1^Research Unit for Orthopaedic Sports Medicine and Injury Prevention, Institute for Sports Medicine, Alpine Medicine & Health Tourism, UMIT - Private University for Health Sciences, Medical Informatics and Technology, Hall, Austria; ^2^Department of Sports Medicine, Exercise Physiology and Prevention, Centre for Sport Science and University Sports, University of Vienna, Vienna, Austria

**Keywords:** muscle remodeling, matrix metallopeptidase, exercise training, aging, diabetes, fibrosis, connective tissue, gene expression

## Abstract

Skeletal muscle represents the largest body-composition component in humans. In addition to its primary function in the maintenance of upright posture and the production of movement, it also plays important roles in many other physiological processes, including thermogenesis, metabolism and the secretion of peptides for communication with other tissues. Research attempting to unveil these processes has traditionally focused on muscle fibers, i.e., the contractile muscle cells. However, it is a frequently overlooked fact that muscle fibers reside in a three-dimensional scaffolding that consists of various collagens, glycoproteins, proteoglycans, and elastin, and is commonly referred to as extracellular matrix (ECM). While initially believed to be relatively inert, current research reveals the involvement of ECM cells in numerous important physiological processes. In interaction with other cells, such as fibroblasts or cells of the immune system, the ECM regulates muscle development, growth and repair and is essential for effective muscle contraction and force transmission. Since muscle ECM is highly malleable, its texture and, consequently, physiological roles may be affected by physical training and disuse, aging or various diseases, such as diabetes. With the aim to stimulate increased efforts to study this still poorly understood tissue, this narrative review summarizes the current body of knowledge on (i) the composition and structure of the ECM, (ii) molecular pathways involved in ECM remodeling, (iii) the physiological roles of muscle ECM, (iv) dysregulations of ECM with aging and disease as well as (v) the adaptations of muscle ECM to training and disuse.

## Introduction

Skeletal muscle is an important body-composition component in humans, typically accounting for more than 40% and 30% of total body mass in men and women, respectively (Kim et al., 2002). The most apparent function of skeletal muscles is to generate the forces required to maintain an upright posture and produce movement. However, skeletal muscles do also play important roles in many other physiological processes, including thermogenesis (Rowland et al., 2015), metabolism (Baskin et al., 2015) and the secretion of numerous peptides for communication with other tissues (Pedersen and Febbraio, 2012). Thus, the promotion and maintenance of skeletal muscle health is of vital importance. Although, in recent years, pharmacological exercise mimetics have attracted increasing scientific interest (Fan and Evans, 2017), it is still physical exercise that is considered the by far most potent and universally applicable tool for these purposes.

Over the past decades, thousands of training studies have been performed in an attempt to identify the exercise modalities most suited to increase muscle size and improve its functional characteristics in different cohorts (for instance, at the time this manuscript was written, Pubmed yielded more than 24,000 results for the search operators “exercise” and “muscle strength”). The outcomes of these studies have inspired various exercise prescription guidelines, probably the best known of which are the position stands published and updated in irregular intervals by the American College of Sports Medicine (2009), Garber et al. (2011). Most studies base their evaluation of the efficacy of training interventions on the examination of contractile muscle cells. Frequently studied parameters involve muscle size as measured at the organ (Fisher et al., 2011) or cellular level (Schoenfeld, 2010), fiber type distribution (Adams et al., 1993), architecture (Aagaard et al., 2001) as well as neural drive to muscles (Folland and Williams, 2007).

The wealth of information on the malleability of skeletal muscles notwithstanding, it is a frequently overlooked fact that muscle fibers are embedded into an extracellular matrix (ECM) consisting of a mesh of collagenous components as well as a mixture of further macromolecules, such as various glycoproteins and proteoglycans. Recent research has demonstrated that the ECM plays an important role in the development (Thorsteinsdóttir et al., 2011), growth (Fry et al., 2017) and repair of muscles (Calve et al., 2010) as well as the transmission of contractile force (Street, 1983). While evidence to demonstrate the malleability of the ECM exists, only a paucity of studies has reported its reactions to different forms of training, suggesting that the physiological role of the ECM is not yet fully appreciated by exercise specialists. Aiming to stimulate further research into the training responses of the non-contractile components of skeletal muscles, we provide an overview over the current state of knowledge concerning the composition, structure and regulation of the ECM, its physiological roles, dysregulations associated with aging and metabolic disorders as well as adaptations to physical exercise.

## Composition and Structure of Skeletal Muscle ECM

The ECM of skeletal muscles is a complex meshwork consisting of collagens, glycoproteins, proteoglycans, and elastin (Takala and Virtanen, 2000; Halper and Kjaer, 2014). Collagens form a network of intramuscular connective tissue (IMCT), i.e., the central, fibrous components of the ECM. The IMCT is typically depicted to be organized in three layers: (i) the endomysium, representing the innermost layer that encloses individual muscle fibers, (ii) the perimysium bundling groups of muscle fibers, and (iii) the epimysium enveloping the entire muscle. The great structural complexity of the IMCT network evidenced by scanning electron micrographs suggests that this traditional classification may be simplistic and that a higher order organization of muscle ECM yet needs to be defined (Gillies and Lieber, 2011). Research into fascial tissues further considers the layers of IMCT as part of a complex system of interconnected and interwoven connective tissues that *“surrounds, interweaves between, and interpenetrates all organs, muscles, bones and nerve fibers, endowing the body with a functional structure, and providing an environment that enables all body systems to operate in an integrated manner”* (Adstrum et al., 2017; Stecco et al., 2018). This system, which is commonly referred to as fascial system, is increasingly recognized as important target in sports medicine (Zügel et al., 2018).

**FIGURE 1:**
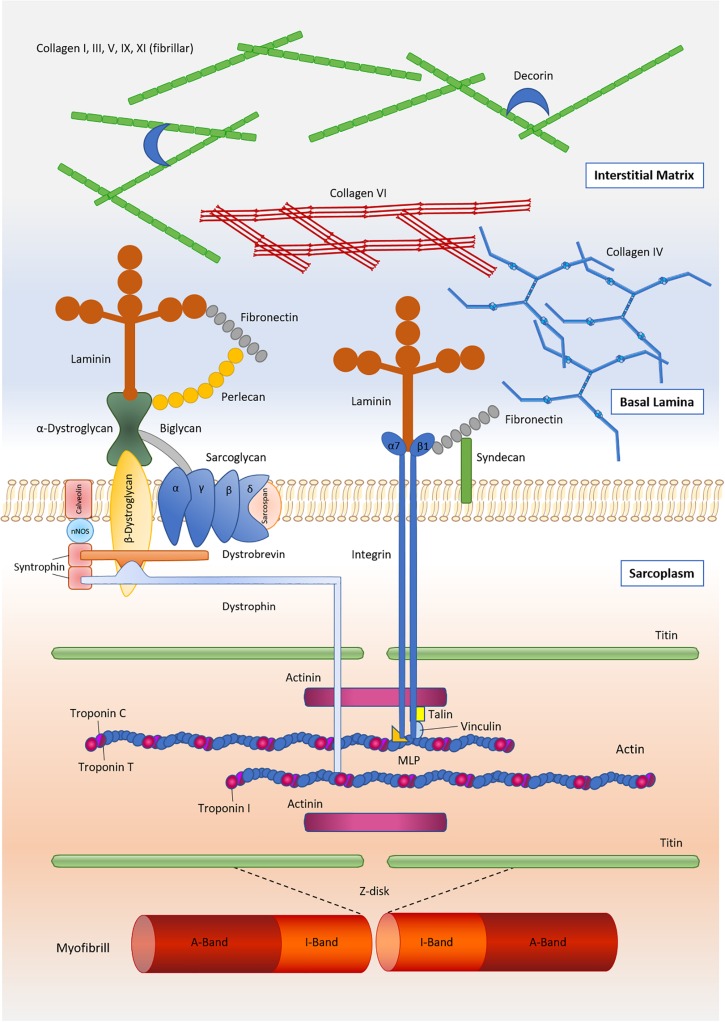
Main components of the skeletal muscle extracellular matrix and its linkage to the contractile components of muscle.

The IMCT contains various forms of collagens with types I and III being most abundant (Duance et al., 1977; Light and Champion, 1984; Gillies and Lieber, 2011; McKee et al., 2019). The endomysium interfaces with the myofiber sarcolemma at a specialized basement membrane, which consists primarily of type IV collagen and laminin (Sanes, 1982; Martin and Timpl, 1987; Kjaer, 2004). The concentration of these two components has been found to differ in dependency of muscle fiber type, with slow twitch fibers featuring substantially greater concentrations of collagen IV but lower concentrations of laminin (Kovanen et al., 1988). Laminin, in turn, serves as ligand for two sarcolemmal receptors – the dystrophin-associated glycoprotein complex and the α7β1 integrin (Grounds et al., 2005) – located at costameres, which are membrane-bound protein structures aligned in register with the Z-disks of myofibrils. Integrins are thought to act in a bidirectional manner, allowing intracellular signaling molecules to regulate external adhesion (“Inside-Out” signaling), and transferring external stimuli to affect cellular processes (“Outside-In” signaling) (Boppart and Mahmassani, 2019). Thereby, cytoskeletal sheer stress induces the intracellular binding of proteins such as talin, vinculin or kindlin, leading to a conformation change of the integrin receptor and allowing the extracellular domains of the receptor to extend toward proteins within the ECM. In addition, integrin ligands from the extracellular space such as laminin, collagen or fibronectin facilitate the formation of a high-affinity upright state, leading to increased binding to ECM proteins and to integrin clustering especially along focal adhesion complexes (Boppart and Mahmassani, 2019). The dystrophin-associated glycoprotein complex is another important factor in providing a mechanical linkage between the contractile components of skeletal muscle (i.e., actin) and the interconnected layers of the IMCT (Ervasti, 1993; Peter et al., 2011). The main components linking the contractile elements of the muscle to the interstitial matrix are shown in Figure 1.

The collagen superfamily contains a total of 28 different members, of which types I, III, IV, V, VI, XII, XIII, XIV, XV, XVIII, and XXII have been shown to be present in mature skeletal muscle at the gene and/or protein level (Table 1). The fibril-forming types I and III are by far most abundant, with proteomic studies suggesting that they jointly account for approximately 75% of total muscle collagen (McKee et al., 2019). The strong parallel fibers of type I collagen, which are present in the endo-, peri-, and epimysium, are assumed to confer tensile strength and rigidity to the muscle, whereas type III collagen forms a loose meshwork of fibers that bestows elasticity to the endo- and perimysium (Kovanen, 2002). Collagen type IV, a helical molecule, produces a network structure that constitutes the basis of the basal lamina (Sanes, 2003). Collagen type VI has been detected in the epimysial, perimysial, and endomysial interstitium, but in particular in the neighborhood of the basement membrane, where it interacts with the carboxyl-terminal globular domain of type IV collagen (Kuo et al., 1997). Interestingly, collagen VI possesses untypical non-collagenous regions forming a distinct microfibrillar network in most connective tissues (Maaß et al., 2016). Collagen VI mutations result in disorders with combined muscle and connective tissue involvement, including Ullrich congenital muscular dystrophy, Bethlem myopathy, the autosomal dominant limb-girdle muscular dystrophy and the autosomal recessive myosclerosis (Bushby et al., 2014).

**TABLE 1:** Overview of collagenous components of the skeletal muscle extracellular matrix.

Gene(s)	Skeletal muscle RNA expression (protein expression myocytes)*	Protein	Form	Appearance	Role	Source
COL1A1-2	1: 11.0 NX (not detected) 2: 12.1 NX (low)	Collagen type I alpha 1 and 2 chains	Fibrils	Endo-, peri-, and epimysium	Forms strong parallel fibers, confers tensile strength and rigidity	Kovanen (2002)
COL3A1	14.5 NX (not detected)	Collagen type III alpha 1 chain	Fibrils	Endo- and perimysium, myotendinous junction	Forms a loose meshwork of fibers, confers compliance	Kovanen (2002)
COL4A1-6	1: 24.6 NX (not detected) 2: 26.3 NX (high) 3: 17.8 NX (not detected) 4: 7.5 NX (no data) 5: 4.8 NX (no data) 6: 1.0 NX (no data)	Collagen type IV alpha 1-6 chains	Helices	Basal lamina	Produces a network structure, constitutes the basis of the basal lamina	Sanes (2003)
COL5A1-3	1: 5.0 NX (not detected) 2: 3.7 NX (no data) 3: 15.4 NX (medium)	Collagen type V alpha 1-3 chains	Fibrils	Endomysium	Control of collagen fibrillogenesis	Kovanen (2002)
COL6A1-6	1: 20.0 NX (not detected) 2: 30.6 NX (not detected) 3: 31.8 NX (not detected) 4: no data 5: 0.2 NX (low) 6: 0.0 NX (medium)	Collagen type VI alpha 1-6 chains	Beaded filaments	Endo-, peri-, and epimysium (α6-chain) Basal lamina (α3-chain) Myotendinous junction (α5-chain)	Interacts with a large number of molecules and cell surface receptors, maintains muscle functional integrity. Mutations associated with fibrosis and Ullrich, Bethlem or Myosclerosis myopathies	Bönnemann (2011), Sabatelli et al. (2012), Cescon et al. (2015)
COL12A1	21.6 NX (medium)	Collagen type XII alpha 1 chain	FACIT	Endo- and perimysium, myotendinous junction	Linkage protein between fibrillar collagens and other ECM components	Jakobsen et al. (2017)
COL13A1	2.7 NX (not detected)	Collagen type XIII alpha 1 chain	MACIT	Neuromuscular junction	Regulation and formation of neuromuscular synapse. Lack associated with myasthenia	Härönen et al. (2017), Heikkinen et al. (2019)
COL14A1	7.6 NX (not detected)	Collagen type XIV alpha 1 chain	FACIT	Endo- and perimysium, myotendinous junction	Linkage protein between fibrillar collagens and other ECM components. Increases following training at myotendinous junction (protection against strain injury?)	Jakobsen et al. (2017)
COL15A1	12.8 NX (low to medium)	Collagen type XV alpha 1 chain	Multiplexin	Basement membrane	Stabilizes muscle cells and microvessels. Guides motor axons toward muscle targets. Deficiency increases vulnerability to exercise-induced muscle injury and leads to mild forms of myopathies	Eklund et al. (2001), Guillon et al. (2016)
COL18A1	8.1 NX (not detected)	Collagen type XVIII alpha 1 chain	Multiplexin	Basement membrane	May bind growth factors. May link the basement membrane to other basement membrane glycoproteins and endomysium	Gillies and Lieber (2011), Heljasvaara et al. (2017)
COL19A1	3.7 NX (low)	Collagen type XIX alpha 1 chain	FACIT	Basement membrane	Presence at early embryonic stages is relevant for the muscle tissue differentiation. Acts as a cross-bridge between fibrils and other extracellular matrix molecules	Khaleduzzaman et al. (1997), Sumiyoshi et al. (2001)
COL22A1	0.5 NX (not detected)	Collagen type XXII alpha 1 chain	FACIT	Myotendinous junction	Integrates ECM and contributes to mechanical stability of the myotendinous junction. Knockdown of COL22A1 results in dystrophy-like muscle phenotype in zebrafish	Koch et al. (2004), Charvet et al. (2013)

*RNA expression summary shows the consensus RNA-data based on normalized expression (NX) data and protein expression comprises profiles using single as well as independent antibodies directed against different, non-overlapping epitopes on the same protein from the Human Protein Atlas (http://www.proteinatlas.org, Uhlen et al., 2010). FACIT, Fibril Associated Collagen with Interrupted Triple Helices; MACIT: Membrane Associated Collagen with Interrupted Triple Helices. Multiplexin: Collagen with Multiple Triple Helix Interruptions.

Collagen types XII, XIV, XIX, and XXII belong to the Fibril Associated Collagens with Interrupted Triple helices (FACIT; Chiquet et al., 2014; Calvo et al., 2020), whereby collagen type XXII seems to be expressed exclusively at tissue junctions such as the myotendinous junction in skeletal and heart muscle (Koch et al., 2004).

Bioinformatic tools to screen the human proteome of normal and diseased tissues allowed to characterize the global composition of the ECM proteome, or “matrisome.” In total, 1,027 genes have been linked to the ECM, whereby core matrisome proteins (ECM glycoproteins, collagens, and proteoglycans) could be distinguished from matrisome-associated proteins (ECM-affiliated proteins, ECM regulators, and secreted factors that may interact with core ECM proteins) (Naba et al., 2016). Given the complexity of human skeletal muscle tissue involving multinucleated muscle fibers, immune cells, endothelial cells, muscle stem cells, non-myogenic mesenchymal progenitors, and other mononuclear cell (Bentzinger et al., 2013a), future research would be needed to elucidate the contribution of each of these cells to the structure and remodeling of the IMCT. Gene signatures derived, e.g., from RNA-seq of isolated muscle fibers and other cell types comprise a promising tool in the deconvolution of bulk skeletal muscle tissue (Rubenstein et al., 2020).

## Physiological Regulation of ECM Genes

The homeostasis of the ECM is maintained through finely tuned anabolic and catabolic processes that are governed by various growth factors, proteoglycans and enzymes responsible for collagen degradation. After binding to membrane-bound receptors, growth factors belonging to the transforming growth factor beta (TGF-β) superfamily have been found to induce the phosphorylation of Smad proteins that transduce extracellular signals to the nucleus where they activate downstream gene transcription resulting in collagen production (MacDonald and Cohn, 2012). Another, albeit less described, factor of similar function is the connective tissue growth factor (CTGF), overexpression of which has been reported to provoke dystrophy-like muscle fibrosis and functional deficits (Morales et al., 2011).

The function of these anabolic factors is mostly regulated by small leucine-rich proteoglycans (SLRPs). Decorin, the prototype member of this family, deactivates the profibrotic TGF-β and CTGF (Zhu et al., 2007; Brandan and Gutierrez, 2013) and also limits fibrillogenesis by directly binding to type I collagen (Reese et al., 2013). Another SLRP is represented by biglycan, which competes with decorin for the same binding site on collagen (Schönherr et al., 1995) and is likely to play a role in both muscle formation and regeneration (Brandan et al., 2008).

Transcriptional regulation of protein formation seems to be an important factor in ECM plasticity. In this respect, it has been shown that protein expression in skeletal muscle is weakly regulated at the mRNA level leading to big differences in mRNA and protein abundance in various tissues (Wang et al., 2019). Interestingly, the pattern of protein regulation depends on protein function, whereby the association between mRNA and protein is higher for ECM and collagen fibril organization (Makhnovskii et al., 2020). Another interesting aspect in the regulation of the amount of ECM proteins is the fact that induction of transcription seems to be rather slow for collagen as it takes almost 3 days to fully induce transcription. In contrast secretion rates are adapted quickly as they are elevated in less than 1 h. In high collagen producing cells, the pathway is controlled by post-transcriptional regulation which requires feedback control between secretion and translation rates (reviewed in Schwarz, 2015).

With respect to tissue remodeling, two families of enzymes, matrix metalloproteinases (MMPs) and tissue inhibitors of metalloproteinases (TIMPs), are involved in the regulation of ECM homeostasis. MMPs are proteolytic enzymes that degrade various types of collagens and are inhibited by TIMPs (Visse and Nagase, 2003; Alameddine, 2012). Specifically, MMP-1 and MMP-8 initiate the degradation of collagens I and III (prevalent in endo-, peri-, and epimysium), whereas MMP-2 and MMP-9 break down type IV collagen (the major collagenous component of the basement membrane) (Corcoran et al., 1996). TIMP-1, -2, and -4 are capable of inhibiting all known MMPs (Christensen and Purslow, 2016).

## ECM and Skeletal Muscle Force

The interaction of actin and myosin as well as many other sarcomeric proteins results in shortening of muscle fibers. Traditional biomechanical models often depict muscle-tendon units as systems, in which the forces generated through fiber shortening are transmitted longitudinally along the muscle fiber and further, at the myotendinous junction, onto the tendon. Close to the myotendinous junction, myofibers feature finger-like processes, which are made from invaginations of the plasma membrane (Knudsen et al., 2015). This structure increases the surface area available for force transmission. Force transmission is expected to occur between the finger-like processes of the muscle fiber and collagen fibers located within the invaginations through shearing of the basal lamina (Huijing, 1999). The collagens contained here are types XXII, which forms an inner layer, as well as III, VI, XII, and XIV, which lie further away from the muscle fiber membrane (Jakobsen et al., 2017). Although its precise role is still unclear, it is interesting to note that in muscles collagen XXII is exclusively located at the myotendinous junction. In zebra fish, deficiency of collagen XXII has been found to result in muscle dystrophy (Charvet et al., 2013), suggesting that this collagen may serve to maintain the structural integrity and stabilize the myotendinous junction.

Considering the fact that a significant portion of fibers in long muscles terminate intrafascicularly without directly reaching a tendon (Barrett, 1962; Hijikata et al., 1993), however, it is clear that the myotendinous pathway cannot represent the only mechanism of force transfer. Intrafascicularly terminating fibers must rely on a medium arranged in parallel with them to transmit their forces onto the passive components of the locomotor system (Sheard, 2000). As first recognized by Street (1983), it is the network of IMCT within the ECM that facilitates such lateral transfer of contractile force. Force transmission across the IMCT network occurs from contractile proteins across costameres to the endomysium (Bloch and Gonzalez-Serratos, 2003; Peter et al., 2011) – as modeling studies suggest through shearing (Sharafi and Blemker, 2011; Zhang and Gao, 2012) – and further to the perimysium which finally merges with aponeuroses and tendons (Passerieux et al., 2007). The first information about the proportions of longitudinal and lateral force transfer in striated muscle stems from elegant experiments by Huijing et al. (1998). After severing the direct connections of multiple heads of the rat extensor digitorum longus muscle, corresponding to 55% of the total muscle mass, from the joint tendon, Huijing et al. (1998) observed that force was maintained at 84% of that of intact muscle. More recently, Ramaswamy et al. (2011) used a yoke apparatus to directly measure the forces transmitted via the longitudinal and lateral pathway. Their results not only confirmed that more than 50% of force was transmitted laterally but also showed that lateral force transfer was significantly reduced in both dystrophic and old rodents. Their results were later confirmed by Zhang and Gao (2014).

Several arguments suggest that the lateral transmission of force is a biomechanical necessity to maintain muscle integrity and improve contraction efficiency. First, it helps to distribute contractile forces over the entire surface of myofibers, which reduces mechanical stress and protects fibers from overextension. This may be particularly important in fiber end regions, which are usually tapered and therefore ill-suited to tolerate excessive forces (Monti et al., 1999). Indirect support for this hypothesis is provided by studies in older subjects (Hughes et al., 2016) or patients suffering from Duchenne dystrophy (Virgilio et al., 2015), in whom dystrophin (i.e., a costameric protein that establishes the mechanical connection between cytoskeleton, sarcolemma and ECM and, thus, facilitates lateral force transmission) is either lost or impaired and the susceptibility for muscle strain injuries is increased.

Also, lateral force transfer is thought to bridge fibers contracting either at different times or to unequal extents (Yucesoy et al., 2006), which would help maintain fiber alignment and, thus, the muscle’s structural integrity (Purslow, 2002). Recently, Dieterich et al. (2017) compared the onset of contraction as determined by electromyography and M-mode ultrasound imaging. Counterintuitively, the authors found the motion onset to precede the electromyography signal in ∼20% of trials, which might be explained by lateral force transfer. Indeed, while longitudinal transmission of forces may be delayed by the need to tauten the elastic elements placed in series with the muscle (Nordez et al., 2009), the translaminar shear linkage between muscle fibers and the IMCT network may allow for immediate force transmission. Finally, lateral force transmission provides a mechanism whereby force may still be generated and transmitted from muscle fibers that are interrupted due to microtrauma or during muscle growth (Purslow, 2010).

In addition to its role in the lateral transfer of contractile force, the ECM may also affect muscle fiber shortening. The contractility of myofibers is often assumed to be constrained by the geometry of its constituting sarcomeres: Sarcomere and, thus, fiber shortening stops when *z*-bands come in contact with myosin filaments. However, these ideas consider only the behavior of the sarcomere as an independent actuator. Under *in vivo* conditions, muscle fibers are embedded into the IMCT network which may interfere with fiber shortening. Indeed, the constant volume principle (Baskin and Paolini, 1967) dictates that during shortening muscle fibers must undergo radial expansion, which has long been experimentally confirmed even at the sarcomeric level (Brandt et al., 1967). Novel computational models and *in situ* measurements in frog muscles by Azizi et al. (2017) have demonstrated that muscle shortening is hindered when radial expansion is limited through physical constraints. Hence, changes in the amount and mechanical properties of the IMCT network into which muscle fibers are embedded may directly affect skeletal muscle contractility. Such a scenario may be represented by muscle fibrosis (Gillies et al., 2017).

## ECM in Skeletal Muscle Development, Growth, and Repair

Apart from force transfer, the skeletal muscle ECM fulfills several important functional roles. Apparently, the IMCT network provides mechanical support to muscle fibers as well as the nerves and blood vessels supporting them. Blood capillaries run in the interstices occupied by endomysium, with their number and density being contingent upon muscle fiber size (Janácek et al., 2009). In addition to this most obvious role, the interaction between myoblasts, differentiated muscle fibers and ECM components is of central importance for the embryogenic development, further growth, and repair of muscle tissue.

The cellular source of the collagenous components of muscle ECM are dedicated IMCT fibroblasts, which originate from different embryogenic sources, including the somites (Nowicki et al., 2003), the lateral plate mesoderm (Pearse et al., 2007) and the neural crest cells (Olsson et al., 2001). As they produce not only fibroblasts but also adipogenic cells, IMCT fibroblasts may be considered as fibroadipogenic progenitors (Uezumi et al., 2010). Recent research has provided evidence that, in addition to these obvious roles, IMCT fibroblasts and the connective tissues produced by them influence both myogenesis (i.e., the formation of muscle progenitors and their differentiation into multinucleate myofibers) and muscle morphogenesis (i.e., the process in which myofibers are assembled into muscles), thus acting as important regulators of muscle development. These complex regulatory processes occurring during embryogenic development are not covered in detail here, but have been extensively reviewed elsewhere (Nassari et al., 2017; Sefton and Kardon, 2019). In brief, the IMCT guides muscle progenitors to their designated target regions, through a combination of attractive (Hepatocyte Growth Factor, Stromal Cell-Derived Factor) and repulsive signals (Ephrin) (Dietrich et al., 1999; Swartz et al., 2001). Through a myriad of transcription factors expressed in IMCT fibroblasts, the IMCT then promotes the proliferation, survival and differentiation of neighboring myoblasts into mature myofibers (Kardon et al., 2003; Hasson et al., 2010; Iwata et al., 2013; Vallecillo-García et al., 2017). Thus, it may be speculated that the IMCT serves as a mesodermal prepattern that controls the sites of myofiber differentiation and, consequently, the ultimate position, size, and shape of muscles.

As post-mitotic tissues, skeletal muscles depend on satellite cells to adapt and regenerate throughout life. These stem cells reside in specialized niches between the sarcolemma of muscle fibers and their encapsulating basement membranes. Satellite cell maintenance, activation and differentiation are governed by complex cascades of transcription factors. For an extensive review of these cellular circuitries, readers are referred to the recent review by Almada and Wagers (2016). Of particular relevance to this manuscript, a growing body of evidence suggests that satellite cell fate is also strongly influenced by the interactions with the ECM niche in which they reside. Indeed, as a dynamic environment, the stem cell niche transmits mechanical and chemical signals that act to protect quiescent stem cells or induce activation, proliferation, and differentiation.

In the quiescent state, satellite cells express the canonical cell regulator paired box protein 7 (PAX7) (Olguin and Olwin, 2004). *In vitro* studies have demonstrated that a greater portion of satellite cells express PAX7 when cultured on matrigel, a mixture of ECM proteins and growth factors (Wilschut et al., 2010; Grefte et al., 2012). Further support for the notion that the ECM is actively involved in the maintenance of satellite cell quiescence comes from reports that satellite cells removed from their niche quickly enter the cell cycle and lose their capacity for myogenic differentiation (Gilbert et al., 2010). Intriguingly, satellite cells appear to also be able to sense and respond to different ECM mechanical properties. In fact, PAX7 expression and satellite cell survival are greater when cultured on hydrogels that mimic the physiological stiffness of muscle (Gilbert et al., 2010). Also, satellite cells cultured on soft hydrogel feature greater functional capacity after transplantation into recipient muscle (Cosgrove et al., 2014).

In addition, ECM components have been shown to influence stem cell division. Specifically, the proteins fibronectin (Bentzinger et al., 2013b) and collagen VI (Urciuolo et al., 2013) as well as the proteoglycans syndecan 3, syndecan 4, perlecan, and decorin (Cornelison et al., 2001; Brack et al., 2008) have been identified as the niche constituents influencing the balance between differentiation and self-renewal and, thus, the maintenance of skeletal muscles’ regenerative capacity.

Upon muscle trauma or in response to increased loading, the usually mostly quiescent satellite cells become activated and differentiate into myoblasts to finally fuse into mature myofibers. While this process requires the timely expression of various transcription factors, such as myogenic factor 5, myogenic determination protein or myogenin (Almada and Wagers, 2016), several studies point to the influence of the ECM on each of these steps. Experiments with mouse (Grefte et al., 2012) or porcine myoblasts (Wilschut et al., 2010) have shown that myoblast fusion is positively influenced by matrigel but not by single substrates present in the ECM niche. The contributions of single proteins are still poorly understood, however, the concomitant presence of poly-D-lysine and laminin (Boonen et al., 2009), glycosaminoglycans (Rønning et al., 2013), and heparin sulfate proteoglycans (Gutiérrez and Brandan, 2010) appear to play a prominent role in satellite cell proliferation and differentiation. Upon activation of skeletal muscle stem cells, local remodeling of the ECM is accompanied by the deposition of laminin-α1 and laminin-α5 into the basal lamina of the satellite cell niche (Rayagiri et al., 2018). In mice, it has been shown that muscle satellite cells produce ECM collagens to maintain quiescence in a cell-autonomous manner with collagen V being a critical component of the quiescent niche, as depletion leads to anomalous cell cycle entry and gradual diminution of the stem cell pool (Baghdadi et al., 2018). Just as for the maintenance of quiescence, adequate mechanical properties of the ECM niche may also be important for satellite cell maturation. Indeed, myotubes have been found to differentiate optimally on substrates with muscle-like stiffness (Engler et al., 2004). Jointly, these data suggest that ECM stiffening accompanying both different musculo-skeletal disorders and the aging process may negatively influence a muscle’s regenerative capacity.

## Remodeling of Muscle ECM With Aging

At older age, skeletal muscles typically demonstrate fibrotic morphology (Lieber and Ward, 2013). As opposed to fascial densification, where the general structure of collagens may be preserved (Pavan et al., 2014), age-associated muscle fibrosis is characterized by the loss of the clear two-directional lattice orientation of healthy perimysial collagen fibers and its replacement by an erratic fiber network featuring decreased crimp formation (Järvinen et al., 2002). Also, absolute collagen content and (non-enzymatic) cross-linking of collagen fibers may be increased (Haus et al., 2007b). Thereby, the elastic modulus of the ECM can be increased approximately 35-fold (from ∼12 kPa in young to ∼418 kPa in old muscle; Yin et al., 2013), with this effect being driven by an accumulation of densely packed and extensively cross-linked collagen (Wood et al., 2014). In large-bodied, long-lived animals, such as the Weddell seals, a 35–40% increase in extracellular space has been observed as total and relative collagen contents increase with age. However, this increase is associated with a shift toward a higher ratio of type I to type III collagen (Hindle et al., 2009). Furthermore, collagen type IV concentration is enhanced in the basal lamina of slow twitch muscles, whereas laminin concentration seems to decrease with age (Kovanen et al., 1988). The increased deposition of basal lamina proteins has also been shown to expel satellite cells from their niches, which affects the regulation of satellite cell divisions (Snow, 1977) and may explain the lower numbers of satellite cells typically counted in old as compared to young muscle (Brack et al., 2007). The loss and functional inactivation of stem cells that negatively affects tissue homeostasis can be considered a general hallmark of aging (López-Otín et al., 2013) that must be considered a universal force driving the aging of muscle (Brack and Muñoz-Cánoves, 2016) and other tissues (Oh et al., 2014). In addition to its effects on satellite cells, a dysregulated basal lamina is also expected to disturb the muscle’s regenerative capacity through inadequate support of muscle fibers and disorganized scaffold orientation (Sanes, 2003). A review including an extensive summary of the effects of aging on skeletal muscle ECM has recently been published by Etienne et al. (2020).

Interestingly, data from transcriptional profiling of muscles derived from young and old rats suggest that out of 682 probe sets that differed significantly between young and old animals, 347 genes actually decreased (rather than increased) in aged/sarcopenic muscle relative to young muscles. Of these genes, 24% have been shown to exert a biological role in the ECM and cell adhesion (Pattison et al., 2003). These data support the hypothesis that age-associated changes in the ECM might be driven by a decreased degradation capacity rather than by increased synthesis of collagenous structures. Especially, MMPs seem to play an important role in these processes (de Sousa Neto et al., 2018). This is further supported by findings that suggest a diminished resistance exercise-induced remodeling capacity of ECM structures in aged muscles (Wessner et al., 2019). While the mechanisms are not yet fully understood, these changes are also believed to directly impair muscle function by hindering fiber contractility (Azizi et al., 2017) and lateral force transmission (Sharafi and Blemker, 2011).

## Dysregulation of Skeletal Muscle ECM Consequent to Metabolic Disorders

It is well known that skeletal muscle plays an important role for the insulin-stimulated uptake of glucose (Richter and Hargreaves, 2013). The role of the ECM in this context might be less clear. Muscle-specific integrin β1-deficient mice exhibit a reduction of the insulin-stimulated glucose infusion rate and glucose clearance despite no alterations in food intake, weight, fasting glucose, insulin levels, and GLUT4 protein expression (Zong et al., 2009) suggesting a relationship between aberrant integrin signaling and the development of type 2 diabetes. Furthermore, it has been shown in an animal model of diabetes that impaired insulin sensitivity is associated with reduced protein levels of the Dp427 isoform of dystrophin and the alpha/beta-dystroglycan subcomplex (Mulvey et al., 2005).

Increased amounts of type I and III collagen were found in both type 2 diabetic and also non-diabetic obese subjects (Berria et al., 2006) and overfeeding in humans was associated with increases in the expression of genes associated with the IMCT (collagens I, III, IV, V, SPARC, integrin; Tam et al., 2014) and alterations in gene pathways related to ECM receptor interaction, focal adhesion, and adherens junction (Tam et al., 2017). However, feeding a high-fat diet to mice led to a reduction of COL1, COL3, and COL6 gene expression levels, but not protein levels (Tam et al., 2015).

The degradation of collagens through MMPs has been shown to be an essential constituent of ECM remodeling (Cui et al., 2017). Whether this might also be true in the context of diabetes has been investigated in an animal study. Interestingly, the genetic depletion of MMP9 did not induce insulin resistance in lean mice despite resulting in an increase of collagen IV. However, when mice were fed a high-fat diet the deletion caused a profound state of insulin resistance. These results further strengthen the role of IMCT components in the progress of muscle insulin resistance, especially in a state of overfeeding (Kang et al., 2014).

Finally, hyaluronan, a major constituent of the ECM is increased in high-fat diet-induced obesity in mice. Treatments with PEGPH20, which dose-dependently reduces hyaluronan in muscle ECM is suggested for the treatment of insulin-resistance with a concomitant decrease in fat mass, adipocyte size, as well as hepatic and muscle insulin resistance (Kang et al., 2013).

To summarize, various components of the ECM have been shown to be affected in various stages of diabetes. Studies on whether diabetes is linked to muscle weakness are controversial (Leong et al., 2015; Li et al., 2016) and it remains to be elucidated whether the changes in ECM-related pathways are directly involved in this context.

## Adaptations to Physical Training and Disuse

The first evidence to indicate the malleability of IMCT in response to physical activity was published as early as in the 1970s, when Suominen and Heikkinen (1975) and Suominen et al. (1977) found greater levels of prolyl hydroxylase (an enzyme promoting the biosynthesis of collagen) in endurance-trained athletes as well as, in a longitudinal study, after eight weeks of aerobic training. The effect of endurance exercise on the pro-collagenous enzymatic activity was later found to be more prominent in red as compared to white muscle (Takala et al., 1983). Direct measurements of collagen content first performed in the late 1980s confirmed that the (type IV) collagen content increased in the fatigue-resistant soleus muscle of rats following lifelong endurance training (Kovanen et al., 1988). The exercise-induced increase in collagen notwithstanding, Gosselin et al. (1998) found that the muscle stiffening observed with advancing age could be countered by an endurance exercise intervention, which the authors related to reduced hydroxylysylpyridinoline cross-linking of collagen fibers.

The effects of immobilization on the skeletal muscle ECM are not entirely unequivocal. Early studies by Karpakka et al. (1990, 1991) found both hydroxylase activity and hydroxyproline (an amino acid constituting collagens) content to be reduced in rats. Changes in collagen content in response to short-term immobilization or disuse were later found to be rather small (Savolainen et al., 1988; Haus et al., 2007a), which may be explained by a relatively slow turnover rate. A more recent study, by contrast, found the content of collagen I and the biomechanical properties (elastic modulus, max stress and yield stress) of crural fascia ensheathing the rat triceps surae muscle to be significantly increased after as little as 21 days of hindlimb unloading (Huang et al., 2018). Interestingly, these changes could be prevented through the application of vibration to the rats’ hind paws twice a day. In non-exercising humans, immunohistochemical staining suggested no changes in the density of the collagen I network after 60 days of bed rest. In subjects performing a countermeasure exercise protocol consisting of reactive jumps on a sledge system, by contrast, collagen I immunoreactivity was reduced as compared to baseline levels (Schoenrock et al., 2018).

Yet another model that allows for the adaptability of muscles’ ECM to be studied is functional overload induced by surgical synergist elimination. In one of the first respective studies, Williams and Goldspink (1981) severed the tendons of the plantaris and gastrocnemius muscles of male rats to overload the soleus muscles. The muscle hypertrophy observed three weeks after tenotomy was accompanied by increases in the IMCT concentration (>45%) and the IMCT-to-muscle tissue ratio. Histological analyses further suggested that the increase in IMCT was mostly due to a thickening of the endomysium. Focusing on the myotendinous junction, Zamora and Marini (1988) performed similar experiments and isolated the rat plantaris muscle through tenotomy of the soleus and ablation of the gastrocnemius muscles. In comparison with control animals, the fibroblasts located at the myotendinous junction developed a higher degree of activation of cytoplasm, nucleus and nucleolus after as little as one to two weeks of functional overload. A more recent study tested the effect of IL-6 on overload-induced ECM remodeling by comparing wild-type and IL-6-knockout mice (White et al., 2009). While the gains in myofiber cross-sectional area were similar after 21 days of functional overload, the increases in muscle wet weight were significantly larger in IL-6-knockout mice. Histological analyses confirmed that this surplus gain in muscle weight could be explained by significantly larger increases in non-contractile tissue content and hydroxyproline concentration, which is indicative of collagen content and fibrosis. In agreement with this observation, procollagen-1, IGF-1, and TGF-β mRNA levels were significantly higher in IL-6-deficient mice. Conversely, mRNA expression of MyoD, a transcription factor required for myo- rather than fibrogenic differentiation of satellite cells (Zammit, 2017), was significantly attenuated in animals lacking IL-6. Jointly, these results indicate that synergist elimination induces an increase in IMCT content and, specifically, a thickening of endomysial structures in overloaded muscles. These adaptations may serve to modulate the muscles’ non-contractile structures to increased functional demands. IGF-1 appears to play an important role in the regulation of this process, as lack of IGF-1 has been shown to lead to excessive accumulation of IMCT and, potentially, impaired muscle regenerative potential.

One of the first studies to test and compare different forms of resistance-like exercise in men was performed by Brown et al. (1999) who reported that, following a single bout of concentric contractions, markers of collagen breakdown (hydroxyproline and serum type I collagen) were not increased. By contrast, eccentric muscle action increased serum collagen levels by >40% for up to 9 days post-exercise, indicating that eccentric contractions may be superior in promoting collagen breakdown. These results were confirmed in two later studies similarly using high-intensity eccentric exercise that found both increased procollagen processing and type IV collagen content as well as higher MMP and TIMP activities (Crameri et al., 2004; Mackey et al., 2004). Interestingly, Crameri et al. (2004, 2007) also reported an increase in tenascin C, a glycoprotein present in the ECM that is assumed to direct cell migration following injury, irrespective of whether muscle damage was induced by voluntary or electrically induced muscle damage. The transient upregulation of tenascin C and other ECM glycoproteins (e.g., fibronectin and hyaluronic acid) is usually referred to as the “transient matrix,” the appearance of which is considered an essential first step for successful muscle repair, as it provides important cues driving muscle stem cell regenerative potential (Calve et al., 2010; Tierney et al., 2016). The release of ECM glycoproteins is reportedly accompanied by increased MMP-9 activity in young, but decreased MMP-9 and MMP-15 activity in old subjects (Wessner et al., 2019). These findings suggest that an acute bout of resistance exercise triggers a catabolic response in young muscle but that this effect may be impaired at older age. The subsequent anabolic reaction, characterized by the upregulation of structural collagens (I, III, IV) and laminin, has been found to occur with a significant delay, thus suggesting that muscle repair consequent to an acute bout of damaging (lengthening) contractions follows a biphasic nature (Mackey et al., 2011; Hyldahl et al., 2015). Interestingly, a recent study by Sorensen et al. (2018) found that the appearance of the transient matrix was blunted in physically active old as compared to young subjects. This observation supports the notion that dysregulated ECM cues may be responsible for the increased ECM deposition and reduced stem cell activity typically seen in older muscle (Grounds, 1998).

One of the first studies to directly compare different forms of muscular contraction in terms of their acute ECM remodeling potential was published by Heinemeier et al. (2007). These authors performed a study in rodents and found that the activity of genes associated with collagen biosynthesis (e.g., collagens I and III) as well as growth factors (e.g., TGF-β1) were upregulated after all forms of physical exercise but most prominently so after eccentric training. In humans, collagen protein fractional synthesis rates have also been proposed to be more increased following an acute bout of eccentric as compared to concentric training (Holm et al., 2017), although this notion is not unchallenged (Moore et al., 2005). Jointly, these results suggest that particularly eccentric exercise is a potent stimulus that induces microtrauma and IMCT cell turnover, with the latter assumed to represent the organism’s attempt to prevent the muscle from re-injury (Mackey et al., 2011; Hyldahl et al., 2015; Takagi et al., 2016). In fact, diminished MMP activity after prolonged training consisting of electrically evoked isometric contractions in rats may reflect successful ECM reinforcement (Ogasawara et al., 2014), whereas prolonged increases in MMP- and TIMP-activity in the plantaris muscle of mice following surgical removal of the gastrocnemius and soleus muscle could be indicative of ongoing ECM remodeling (Mendias et al., 2017).

In addition to contraction mode, skeletal muscle ECM may also be sensitive to exercise intensity. Carmeli et al. (2005) tested the effects of treadmill running at either high or low intensity in rats and found that MMP-2 (one of the enzymes responsible for the breakdown of collagen IV mainly present in the muscle’s basement membrane) was increased after high-intensity exercise only. In humans, by contrast, one study by Holm et al. (2010) compared the effects of unilateral knee extension exercise as performed at either low or high (16% or 70% of the individual one-repetition maximum, respectively) intensity, with the number of repetitions adjusted to match the interventions for the total load lifted. In this study, collagen fractional synthesis rates were evenly increased following both interventions.

In terms of ECM adaptations to prolonged resistance training, only data from animal studies exist. de Sousa Neto et al. (2018) reported that 12 weeks of resistance training consisting of ladder climbs with progressive, additional loads equivalent to 65–100% of each individual’s maximum carrying capacity upregulated MMP-2 activity in the plantarflexor muscles of old rats, while down-regulating MMP-2 and MMP-9 in blood circulation. The authors’ conclusion that resistance training might, therefore, be a useful tool to maintain ECM remodeling at older age has recently received empirical support by another training study in rats that used the same training protocol and showed a reduced deposition of connective tissue in trained older muscles (Guzzoni et al., 2018).

To summarize, several studies investigating the acute effects of physical activity in both rodents and men have indicated that exercise may stimulate both the degradation and synthesis of collagen in skeletal muscle. The repair of exercise-induced microtrauma follows a biphasic pattern, in which glycoproteins first create a transitional matrix to guide catabolic processes, and anabolic processes to reinforce the IMCT structure occur with a significant delay. The potential of exercise to induce ECM remodeling seems to be dependent on contraction mode with eccentric contractions triggering a greater response than either concentric or isometric muscle action. Few studies testing the results of different exercise intensities are available, with so-far results suggesting that protein breakdown (but not synthesis) may be provoked more strongly by higher intensities. Disuse acutely downregulates the activity of enzymes related to the biosynthesis of collagens, although at the protein-level changes occur at a slow rate. Cross-sectional comparisons involving (mostly endurance-) trained rodents suggest that chronic physical activity may result in a reinforced IMCT phenotype. The only long-term longitudinal training studies available to date have been performed in rodents and suggest that prolonged resistance training may be useful in countering excessive IMCT accumulation at older age. The physiological and functional consequences of training-induced IMCT remodeling require further investigation.

## Conclusion

The present review aimed to provide an overview over the current state of knowledge concerning the skeletal muscle ECM, which plays an essential, albeit frequently underestimated role in the maintenance of muscle homeostasis, influences muscle function and adaptation and may be a key for the treatment of muscular and metabolic disorders consequent to aging or disease.

As a complex meshwork of various collagens, glycoproteins, proteoglycans and elastin, the ECM embeds contractile muscle fibers and serves via integrins and the dystrophin-associated glycoprotein complex, respectively, as biochemical and mechanical interface between muscle cells and their surroundings. The assembly of its collagenous scaffold is mostly promoted by the growth factors TGF-β and CTGF, which are regulated by different proteoglycans, such as decorin and biglycan. Moreover, proteolytic enzymes (MMPs) as well as their inhibitors (TIMPs) are involved in ECM regulation.

Functionally, the ECM serves as medium for the transmission of contractile force, which may not only serve to increase the efficiency of muscular contraction but also to protect muscle fibers from excessive stress and facilitate healing of microtrauma. In addition to its functional role, the ECM is actively involved in the regulation of the muscle’s pool of satellite cells. ECM niches, established between sarcolemma and basement membrane, protect satellite cell from entering the cell cycle and, thus, help maintain the muscle’s regenerative potential. Specific ECM components, such as fibronectin, collagen VI and different proteoglycans, may additionally promote stem cell division. Conversely, laminin, glycosaminoglycans and other proteoglycans have been shown to promote satellite cell differentiation and their fusion into mature myofibers.

Scientific evidence further demonstrates that the ECM of skeletal muscles is a malleable tissue that may undergo remodeling processes consequent to aging, disease, physical training or disuse. Specifically, aging typically leads to overall increased deposition of collagenous tissue, changes in collagen composition (shift toward higher type I to type III collagen) and increased non-enzymatic collagen cross-linking (through advanced glycation end products). These changes, which are possibly mediated through decreased MMP activity, lead to stiffening of the muscle’s ECM and may impair the muscle’s function and regenerative potential.

Extracellular matrix remodeling may also be associated with metabolic disorders, such as diabetes. Excessive food intake has been found to lead to increased expression of ECM-related genes (collagens I, III, IV, V, SPARC, integrin). In turn, such remodeling may impair integrin signaling, thus reducing insulin sensitivity. Further ECM components potentially representing targets for insulin resistance are hyaluronan, the dystrophin-dystroglycan complex as well as MMP9.

Finally, ECM remodeling may be triggered by physical exercise. While actual training studies are scant, there is evidence to show that exercise may acutely promote both increased collagen synthesis (collagens I, III, TGF-β1) and degradation (MMP2, MMP9). Cross-sectional studies in humans and longitudinal studies in rodents further suggest that such increased collagen turnover may lead to reinforced collagenous structures in chronically trained subjects and prevent excessive collagen deposition (i.e., fibrosis) in elderly muscle. Studies investigating the consequences of prolonged disuse have shown controversial results. While early studies reported decreased hydroxylase activity and hydroxyproline content after short-term immobilization, more recent works found increased collagen I content after 21 days of hindlimb unloading in rats but no change after 60 days of bed rest in humans. Further research and particularly human training studies are required to investigate the influence of different training modalities on ECM structure and composition.

## Author Contributions

RC contributed to the literature research and drafted the manuscript. MG and BW contributed to the literature research and revised the manuscript. All authors have approved the final version of the manuscript and agreed to be accountable for all aspects of the work. All persons designated as authors qualify for authorship, and all those who qualify for authorship are listed.

## Conflict of Interest

The authors declare that the research was conducted in the absence of any commercial or financial relationships that could be construed as a potential conflict of interest.
